# How sexuality is affected and managed in patients under antipsychotic drugs

**DOI:** 10.1192/j.eurpsy.2023.2243

**Published:** 2023-07-19

**Authors:** F. Ribeirinho Soares, B. Mesquita, A. M. Fraga, M. Albuquerque, J. O. Facucho, P. E. Santos, D. E. Sousa, N. Moura, P. Cintra

**Affiliations:** 1 Departamento de Saúde Mental, Hospital de Cascais, Cascais; 2 Departamento de Saúde Mental, Hospital de Cascais, Porto; 3 Departamento de Saúde Mental, Centro Hospitalar Barreiro Montijo, Barreiro, Portugal

## Abstract

**Introduction:**

Sexual dysfunction (SD) is a prevalent side effect of antipsychotic drugs (AP), and it impairs patients’ quality of life. Because of the distress caused by it, it should be borne in mind when prescribed since it is responsible for treatment nonadherence or discontinuation. SD affects about 45- 80% of males and 30-80% of females that take it. In SD, all phases of the sexual response cycle may be compromised.

**Objectives:**

This non-systematic review of the literature aims to better understand the antipsychotic-induced SD and its management to better compliance of AP-treated patients without compromising their quality of life.

**Methods:**

A semi-structured review on PubMed linking SD as a side effect of AP drugs.

**Results:**

All AP drugs can cause SD. It seems related to their mechanism of activity at receptors D2, 5-HT2, α1, H1, and M, which are also involved in sexual function. They do it by diminishing arousal, decreasing libido by blocking motivation and reward system and orgasm indirectly, provoking erectile dysfunction by vasodilatation, and decreasing woman lubrification. Hyperprolactinemia is a significant cause of sexual dysfunctions. Haloperidol, Risperidone, and Amisulpride (prolactin elevating AP) are more likely to cause SD than Olanzapine, Clozapine, Quetiapine, and Aripiprazole (prolactin sparing AP). Although psychotic disorders (Schizophrenia and other psychotic disorders) can impact sexual functioning, according to evidence, there is no denying the role of AP in this issue. Aripiprazole, a D2 partial agonist, has been associated with lower rates of SD and seems to reduce the rates of SD in patients previously treated with other AP. Other AP with the same potential dopamine agonist activity, such as Cariprazine and Brexpiprazole, can probably have the same effect. The management of SD induced by AP drugs should include measuring serum prolactin and modifying risk factors like hypertension, smoking, hyperglycemia, and hypercholesterolemia. In that regard, waiting for spontaneous remission, reducing the dose of the AP prescribed, or switching to Aripiprazole are all viable strategies, if possible. Although the evidence supporting the addition of symptomatic therapies is weak, adding dopaminergic drugs (amantadine, bromocriptine, cabergoline) or drugs with specific effects on sexual functioning (such as phosphodiesterase inhibitors or yohimbine) may be helpful in selected cases.

**Conclusions:**

Although all AP drugs can cause sexual dysfunction, it is difficult to determine its true prevalence accurately. AP-induced sexual dysfunction can adversely affect compliance and is one of the factors that must be considered when selecting treatment. In summarizing, Aripiprazole has shown to be the AP with the most favorable profile concerning SD. Cariprazine and Brexpiprazole, being also D2 partial agonists, may cause less SD.

**Disclosure of Interest:**

None Declared

